# Energy crisis precedes global metabolic failure in a novel *Caenorhabditis elegans* Alzheimer Disease model

**DOI:** 10.1038/srep33781

**Published:** 2016-09-22

**Authors:** Sheng Fong, Emelyne Teo, Li Fang Ng, Ce-Belle Chen, Lakshmi Narayanan Lakshmanan, Sau Yee Tsoi, Philip Keith Moore, Takao Inoue, Barry Halliwell, Jan Gruber

**Affiliations:** 1 Internal Medicine Residency Programme, SingHealth Group, Singapore; 2 NUS Graduate School for Integrative Sciences and Engineering, National University of Singapore, Singapore; 3 Science Division, Yale-NUS College, Singapore; 4 Department of Biochemistry, National University of Singapore, Singapore; 5 Institute of Chemical and Bioengineering, ETH Zurich, Switzerland

## Abstract

Alzheimer Disease (AD) is a progressive neurological disorder characterized by the deposition of amyloid beta (Aβ), predominantly the Aβ_1–42_ form, in the brain. Mitochondrial dysfunction and impaired energy metabolism are important components of AD pathogenesis. However, the causal and temporal relationships between them and AD pathology remain unclear. Using a novel *C. elegans* AD strain with constitutive neuronal Aβ_1–42_ expression that displays neuromuscular defects and age-dependent behavioural dysfunction reminiscent of AD, we have shown that mitochondrial bioenergetic deficit is an early event in AD pathogenesis, preceding dysfunction of mitochondrial electron transfer chain (ETC) complexes and the onset of global metabolic failure. These results are consistent with an emerging view that AD may be a metabolic neurodegenerative disease, and also confirm that Aβ-driven metabolic and mitochondrial effects can be reproduced in organisms separated by large evolutionary distances.

Alzheimer disease (AD) is a neurological disorder characterized by progressive memory impairment and cognitive deficits. The neuropathologic hallmarks of AD are deposition of plaque-forming amyloid beta, predominantly as a 42-amino acid peptide (Aβ_1–42_)^1^, and of tangle-forming tau protein aggregates^2^.

Mitochondrial dysfunction and defective energy metabolism are features consistently found in human AD. Reduced energy production, lower oxygen consumption, and decreased complex I and IV activities have all been observed in AD brains (reviewed by ref. 3). Similar metabolic alterations have been observed in model organisms, including transgenic mice (reviewed by ref. 4) and nematodes overexpressing human Aβ^5^. However, exactly how and at what stage of the disease mitochondrial dysfunction occurs remain incompletely understood.

The nematode worm *Caenorhabditis elegans* has several unique advantages as a model organism for ageing and neurodegenerative diseases (reviewed by ref. 6). Due to its genetic tractability, stereotypical nervous system, rapid life cycle, short lifespan (2–3 weeks) and relatively low cost, *C. elegans* may be employed in ways that would be time, labour and cost prohibitive in mammalian models. A validated model of mitochondrial Aβ toxicity in neurons in *C. elegans* would therefore be valuable as a screening platform for drug discovery to expedite development of future AD therapeutics.

While numerous transgenic *C. elegans* strains have been generated to study AD (see Supplementary Table S1), none of the existing strains has constitutive pan-neuronal Aβ_1–42_ expression or displays an age-related onset of behavioural dysfunction, such as that observed in human AD. We have therefore developed a novel *C. elegans* strain with constitutive pan-neuronal Aβ_1–42_ expression and aimed to clarify the temporal relationships between energy metabolism, mitochondrial dysfunction and neurodegeneration in AD pathogenesis. To facilitate delineation of the temporal sequence of these events and for screening and intervention testing, we aimed at generating a strain that would exhibit progressive neuronal dysfunction and middle-age-onset behavioural phenotypes. To this end, we first constitutively expressed full-length human Aβ_1–42_ peptide in all nematode neurons at different gene doses, and selected a line with late onset behavioural phenotypes. After characterizing the behavioural deficits in the Aβ-expressing nematodes, we then investigated the temporal relationships between energy metabolism, mitochondrial dysfunction and neurodegeneration.

## Results

### Generation of constitutive pan-neuronal Aβ_1–42_ -expressing nematodes

Due to unexpected post-translational N-terminal truncation of full length human Aβ_1–42_, several of the current *C. elegans* AD strains have been shown to accumulate truncated Aβ_3–42_, a form that does not significantly contribute to AD pathology in the human brain^7^. To avoid this issue, we added additional nucleotides (GATGCT) to the 3′ DNA end of the human Aβ_1–42_ minigene sequence. This sequence has been shown by McColl *et al.*^8^ to be processed to generate full length Aβ_1–42_ in *C. elegans* (see Supplementary Fig. S1a). In addition, we optimized the codons in the Aβ_1–42_ minigene for optimal Aβ_1–42_ expression level in nematodes (see Supplementary Fig. S1b).

**Figure 1:**
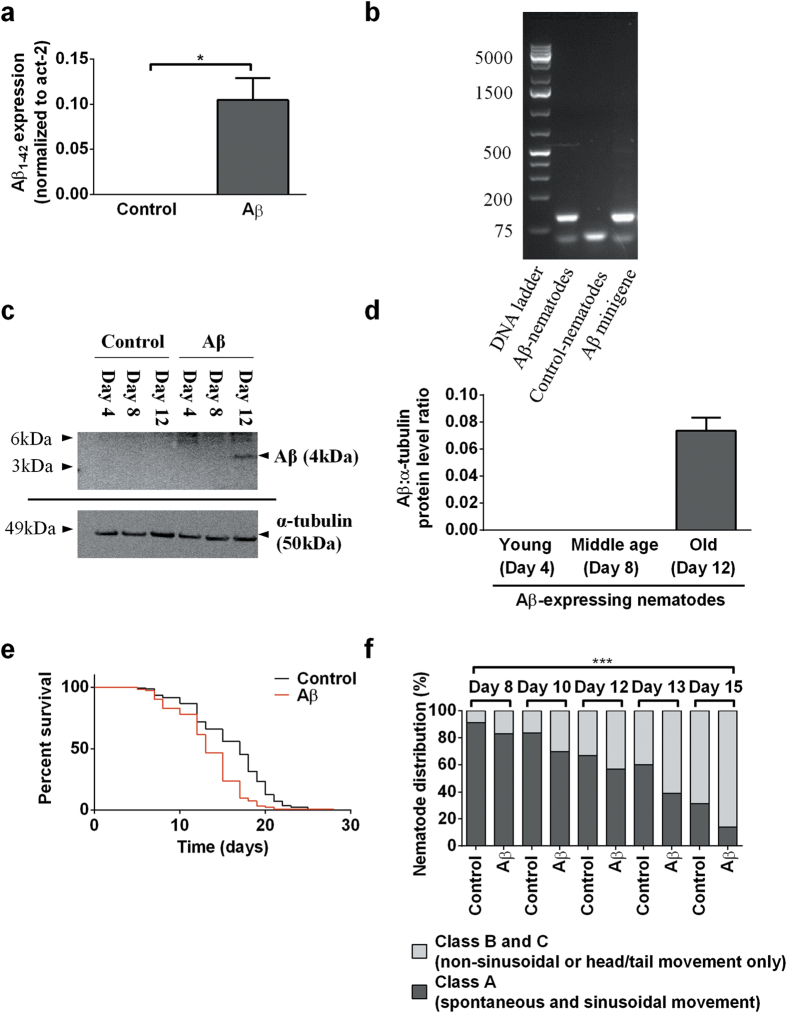
A novel *C. elegans* AD model constitutively expresses low levels of full-length human Aβ_1–42_ and exhibits significantly shorter lifespan and reduced health-span. (**a**) Relative expression levels of human Aβ_1–42_ mRNA in Aβ-expressing and control (day 12) nematodes, normalized to *C. elegans* homolog of human actin (act-2) (***P < 0.001; n = 3 replicates, with 200 nematodes per replicate). (**b**) DNA gel resolving the PCR fragment obtained from amplification of the full length mRNA sequence. The expected full length Aβ band size is 129 bp. (**c**) Immunoblot comparing human Aβ_1–42_ protein levels between Aβ-expressing and control nematodes, in insoluble fractions, at different ages. The expected band size for Aβ and α-tubulin is 4 kDa and 50 kDa respectively. (**d**) Quantification of Aβ band intensity obtained from the Aβ-expressing nematodes of different ages (n = 7 replicates, with approximately 5000 nematodes per replicate). (**e**) Kaplan-Meier survival curves of Aβ-expressing and control nematodes. Aβ-expressing nematodes had a significantly shortened lifespan (log rank test: P < 0.001; n = 122 Aβ-expressing and 168 control nematodes). (**f** ) Aβ-expressing nematodes had normal motility when young, but had a lower percentage of Class A nematodes starting from middle age (day 8) than controls (overall significance by two-way ANOVA: ***P < 0.001; n = 122 Aβ-expressing and 168 control nematodes).

Following the creation of stabilized transgenic nematodes, we performed reverse transcription polymerase chain reaction (RT-PCR) and western blot to confirm the presence of human Aβ_1–42_ mRNA and protein. Using a commercialized Taqman human Aβ gene assay, we first confirmed via real-time PCR that human Aβ_1–42_ mRNA is produced in our Aβ-expressing nematodes but not in the controls (Fig. 1a). To verify that the Aβ_1–42_ mRNA expressed was indeed the full length sequence, we performed another PCR reaction using primers flanking the full length Aβ_1–42_ sequence and showed that the fragment size obtained from the Aβ-expressing nematodes corresponds to that of the human Aβ_1–42_ minigene (Fig. 1b). DNA sequencing of the fragment further confirmed it to be the codon optimized Aβ_1–42_ sequence (see Supplementary Fig. S1c). Immunoblot analysis showed that human Aβ_1–42_ protein was detected only in the insoluble (resolubilised in 5M urea), but not the soluble protein fraction of Aβ-expressing nematodes, suggesting that Aβ is found mainly in aggregated proteins in our transgenic nematodes. Expression of aggregated Aβ protein was only detectable in old nematodes (day 12) but not when they were young (day 4) or middle-aged (day 8; Fig. 1c,d), which correlates to the age-related deterioration in behavioural and biochemical deficits of the Aβ-expressing nematodes. It is important to note that this correlation does not imply any causation. In particular, urea soluble Aβ aggregates themselves are not necessarily responsible for the behavioural and metabolic deficits. Indeed, the observed early metabolic defects themselves might actually interfere with clearance of Aβ and promote accumulation of detectable aggregates. The resultant Aβ-expressing nematodes experienced a significantly shortened lifespan (Fig. 1e, Supplementary Table S2). We also determined nematode health-span by classifying nematodes into motility classes A, B, or C as defined in ref. 9. Compared to controls, Aβ-expressing nematodes showed reduced health-span (Fig. 1f), as indicated by a lower percentage of highly motile (Class A) from day 8 to 15.

### Aβ-expressing nematodes displayed neuromuscular defects and middle-aged onset of behavioural dysfunction

**Figure 2:**
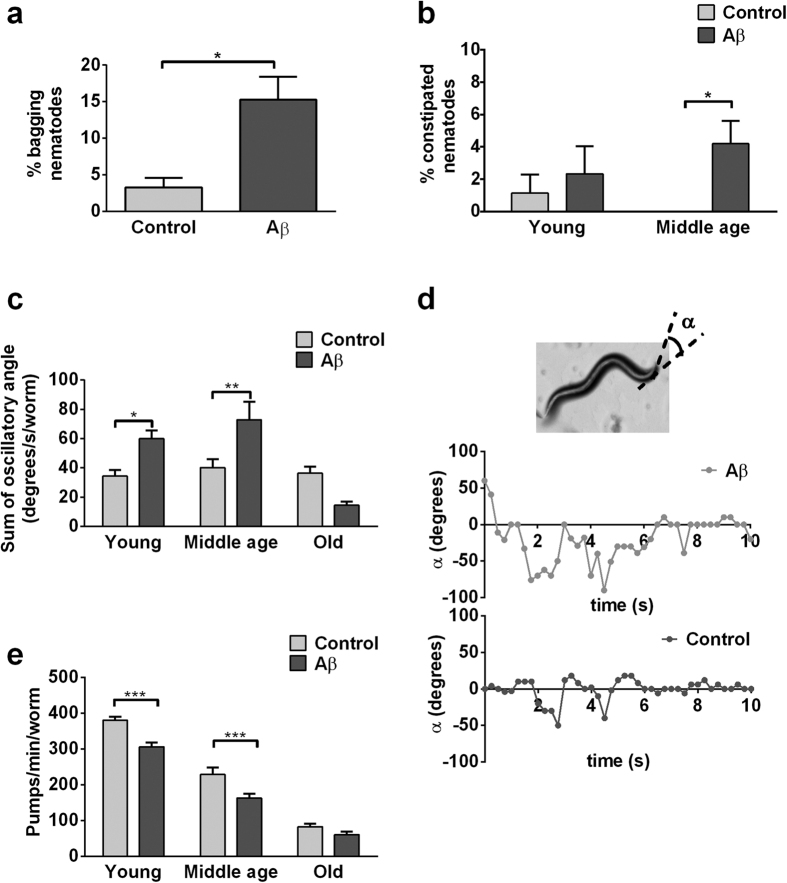
Aβ-expressing nematodes show signs of impaired neuromuscular behaviours. (**a,b**) Compared to controls, there was a greater proportion of bagging (unpaired t-test: *P < 0.05; n = 4 replicates, with 30–40 nematodes per replicate) and constipated Aβ-expressing nematodes (overall significance by two-way ANOVA: *P < 0.05; post-test: middle age, *P < 0.05; n = 4 replicates, with 30–40 nematodes per replicate). (**c**) Aβ-expressing nematodes experienced more severe age-dependent decline in pharyngeal pumping relative to control (overall significance by two-way ANOVA: P < 0.001; post-test: young & middle age, ***P < 0.001; n = 10–15 nematodes). (**d**) Angle of deviation, α, was measured relative to the central axis. Illustrative head shake graphs for middle-aged control and Aβ-expressing nematodes are shown. (**e**) Middle-aged Aβ-expressing nematodes exhibited greater cumulative oscillatory angle (product of angle of deflection and head shake frequency) than controls (overall significance by two-way ANOVA: P < 0.01; post-test: middle age, **P < 0.01; n = 6–11 nematodes). Age classification is as follows: young (day 4 post-hatched), middle-age (day 8 post-hatched), old (day 12 post-hatched).

We further observed numerous signs of neuromuscular defects in the Aβ-expressing nematodes, including higher incidence of internal hatching (bagging^10^; Fig. 2a), defects in defecation that lead to constipation (Fig. 2b), lower rates of pharyngeal pumping (Fig. 2c), and abnormal head shake behaviour (Fig. 2d,e). Bagging may occur rarely in wildtype nematodes as a result of age-related degeneration in the egg laying system, which requires proper formation and functioning of the vulva, vulval muscles and their innervating neurons^11^. Defecation and pharyngeal pumping similarly are neuromuscular events that are tightly regulated and carefully orchestrated by the nervous system^12^,^13^. We also noticed abnormally large oscillatory angle and irregular head shake frequency in the Aβ-expressing nematodes (Fig. 2d). To quantify this behaviour, we calculated the sum of total oscillatory angle as the product between average angle of deflection and head shake frequency. We found a significant difference in this measurement of head shake behaviour in middle-aged Aβ-expressing nematodes compared with controls (Fig. 2e). These abnormal oscillatory head movements, together with the higher incidence of bagging, defects in defecation and in pharyngeal pumping, indicate that neuronal Aβ expression had broad detrimental effects on nematodes’ neuromuscular function.

**Figure 3:**
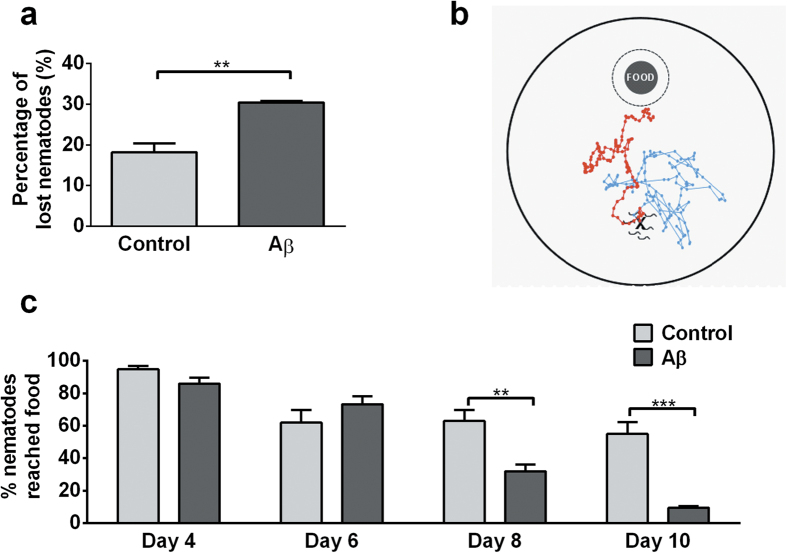
Middle-aged Aβ-expressing nematodes exhibit a pronounced tendency to wander off secondary to deficits in complex sensorimotor function. (**a**) Compared to controls, Aβ-expressing nematodes were twice as likely to wander on food plates (unpaired t-test: **P < 0.01; n = 3 replicates, with 30–40 nematodes per replicate). (**b**) Sensorimotor function was tested using the ‘food race’ assay. Between 100 and 200 nematodes were placed at a starting spot 45 mm away from a food spot and the percentage of nematodes that successfully reached the food was scored after 120 min. (**c**) Compared to controls, Aβ-expressing nematodes were significantly less likely to reach the food, both at day 8 (early middle age) and day 10 (late middle age) (overall significance by two-way ANOVA: P < 0.001; post-tests: day 8, **P < 0.01; day 10, ***P < 0.001; n = 6 replicates, with 100 – 200 nematodes per replicate).

During routine nematode cohort maintenance a certain percentage of nematodes crawl off the side of culture plates and become lost (cannot to be located by the experimenter or found dried at side of plate)^14^. We observed, during our early work with the Aβ-expressing nematodes, that they had a more pronounced tendency to wander off plates than usually seen in controls. To investigate this behaviour further, we quantified nematode loss on fresh food plates and confirmed a significantly higher tendency of Aβ-expressing nematodes to crawl out of the plate (Fig. 3a). To evaluate whether this abnormal wandering behaviour was indicative of a general failure in complex sensorimotor function, we conducted a ‘food race’ assay (Fig. 3b). During this assay, nematodes are placed on a fresh culture plate 45 mm away from a food spot. Nematodes explore the new environment and eventually sense the presence of food and track towards it ref. 15. Aβ-expressing nematodes performed normally in this assay at a young age (day 4 and 6), but were significantly less likely to reach the food at middle age (day 8 and 10, Fig. 3c). This observation reveals an age-related onset of behavioural phenotypes in Aβ-expressing nematodes.

### ATP reduction and ETC complexes dysfunctions precede global metabolic failure in Aβ-expressing nematodes

**Figure 4:**
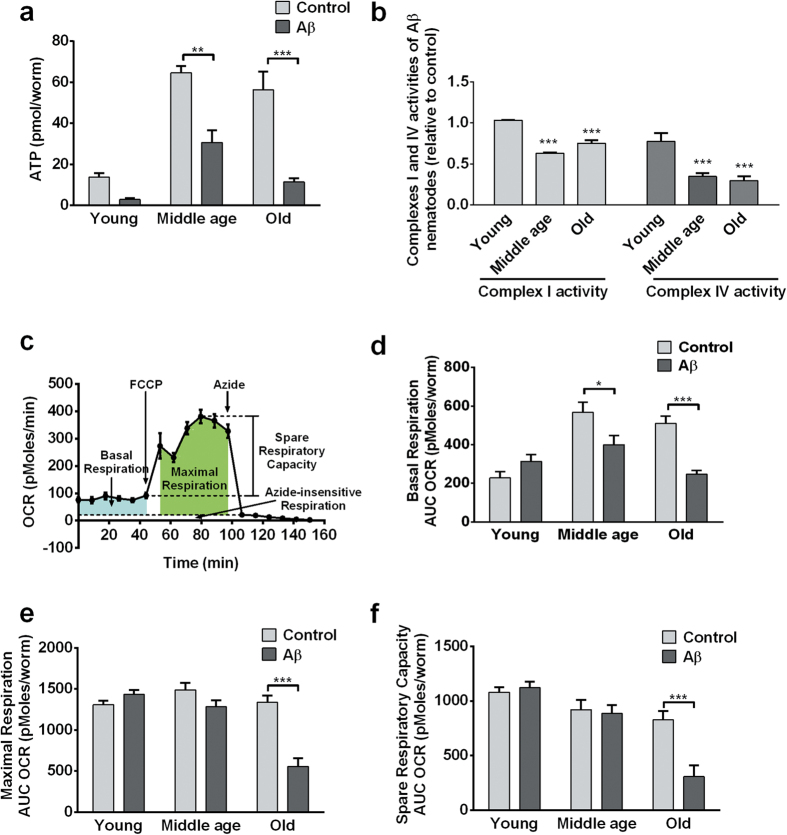
ATP reduction and ETC complex deficits preceded global metabolic failure in Aβ-expressing nematodes. (**a**) Aβ-expressing nematodes had significantly lower ATP levels than controls at middle (**P < 0.01; n = 3 replicates, with 100 nematodes per replicate) and old age (***P < 0.001; n = 4 replicates, with 100 nematodes per replicate). (**b**) Complex I and IV enzymatic activities were significantly lower in middle- and old-aged Aβ-expressing nematodes than in control (***P < 0.001; n = 3 replicates, with 5000 nematodes per replicate). Data were normalized to controls. (**c**) Typical metabolic flux data obtained in *C. elegans*. (**d**) Aβ-expressing and control nematodes had comparable basal respiration when young, but middle- and old-aged Aβ-expressing nematodes showed significantly lower basal respiration relative to control (***P < 0.001; n = 12 replicates, with 10 nematodes per replicate). (**e,f** ) Aβ-expressing nematodes had relatively normal maximal respiration and spare respiratory capacity at young and middle age, but significantly lower spare respiratory capacity and maximal respiration than control at old age (***P < 0.001; n = 12 replicates, with 10 nematodes per replicate).

Upon successful characterization of the age-related behavioural deficiencies in the Aβ-expressing nematode, we proceeded to investigate the temporal relationships between the different components of mitochondrial dysfunction. We measured cellular ATP levels, ETC complex I and IV activities, and metabolic flux as components of mitochondrial functioning. Among the different components, large decreases in ATP level was the earliest event observed in the Aβ-expressing nematodes, even before the onset of behavioural deficit in middle-age. Compared to controls, Aβ-expressing nematodes experienced significantly lower steady-state ATP levels from a young age (Fig. 4a). On the other hand, deficiencies in ETC complex I and IV were observed starting only from middle-age (Fig. 4b).

We further evaluated metabolic flux in nematodes using a Seahorse bioanalyzer, which enables the quantification of basal respiration rate, as well as maximal respiration rate and spare respiratory capacity, in the presence of carbonyl cyanide 4-(trifluoromethoxy) phenylhydrazone (FCCP), a mitochondrial uncoupler, and sodium azide, an ETC complex IV inhibitor (Fig. 4c)^16^. FCCP uncouples oxygen consumption from ATP production by dissipating the proton gradient across the inner mitochondrial membrane, which in turn causes an increase in fuel utilization and oxygen consumption as cells attempt to re-establish the proton gradient^17^. The difference in oxygen consumption during basal state and after FCCP addition thus reveals the spare respiratory capacity of the mitochondria. Sodium azide inhibits most of the cellular respiratory processes and shuts down most oxygen consuming processes. Azide was used to determine baseline drift due to processes that consume oxygen independently of cellular respiration. Finally, the difference in oxygen consumption rate in the presence of FCCP and Azide was used as the maximal oxygen consumption due to cellular respiration. We found that while Aβ-expressing nematodes had comparable basal respiration when young, middle aged, but old-aged Aβ-expressing nematodes showed significantly suppressed basal respiration relative to controls (Fig. 4d). Similarly, Aβ-expressing nematodes had relatively normal spare respiratory capacity and maximal respiration at young and middle age, but significantly lower maximal respiration and spare respiratory capacity at old age (Fig. 4e,f). Given that ATP and ETC complex deficits were detectable in middle-aged Aβ-expressing nematodes, while deficits in mitochondrial maximal respiration and spare respiratory capacity were observable only in old Aβ-expressing nematodes, these data thus suggest that an energy crisis preceded the general metabolic failure in the Aβ-expressing nematodes.

## Discussion

Here we report the first *C. elegans* AD strain designed to constitutively express full-length human Aβ_1–42_ specifically in neurons. The *unc-119* promoter used is well-characterized and highly specific for neurones but we cannot entirely exclude the possibility that some Aβ is also expressed in other tissues. However, most of the phenotypes observed are consistent with predominately neuronal expression. This strain suffered from shortened lifespan and reduced health-span, and exhibited neuromuscular and behavioural dysfunction including internal hatching, constipation, defects in pharyngeal pumping, abnormal oscillatory head movements and middle-age-onset sensorimotor deficits. The middle-age onset and gradual progression of behavioural deficits exhibited by this strain have allowed us to study the temporal relationships between mitochondrial dysfunction and behavioural deficits.

Consistent with a study showing early reduction of ATP levels in pre-symptomatic transgenic AD mice^18^, we found that ATP deficit was an early event experienced by Aβ-expressing nematodes, even before the onset of behavioural deficits and Aβ aggregation. This sequence of events suggests that bioenergetic deficits in the Aβ-expressing nematodes may be independent or causative, rather than as a consequence of aggregation. The large fall in ATP level was followed by dysfunctions in ETC complexes I and IV, which are well characterized features in human AD pathogenesis^4^ that have also been observed in the muscle-specific *C. elegans* strain overexpressing human Aβ^5^. Global metabolic failure, in the form of lower basal and maximal respiration, and decreased spare respiratory capacity, occurred only in old Aβ-expressing nematodes.

The sequence of events suggests that global metabolic failure in Aβ-expressing nematodes is driven by mitochondrial bioenergetic deficits and progressive complex I and IV dysfunctions. Given that 55% of metabolic energy is believed to be used to maintain ionic gradients required to sustain healthy neuronal function, at least in mammals^19^, we believe that the large decrease in ATP level may have also contributed to the behavioural deficits and neuromuscular dysfunctions observed in Aβ-expressing nematodes.

Remarkably, we have detected significant metabolic effects in whole nematodes despite Aβ expression at low levels in the neurons. Our findings confirm earlier findings in a related muscle-specific *C. elegans* strain overexpressing human Aβ^5^,^20^, and support observations previously made in transgenic mice (reviewed by ref. 4) and AD brains (reviewed by ref. 3). This consistency suggests that Aβ-driven metabolic and mitochondrial effects may be a fundamental feature of Aβ toxicity that can be reproduced in organisms separated by large evolutionary distances.

Our findings have provided further evidence to the increasingly popular view that AD may be a metabolic neurodegenerative disease^21^. Aβ has been shown to induce hypometabolism (reduced energy metabolism) *in vitro*^22^,^23^. Proteins involved in energy metabolism have also been found specifically oxidized in the muscle-specific *C. elegans* strain overexpressing human Aβ^24^. There is also substantial evidence from transgenic mice^25^,^26^ and human AD studies that links hypometabolism to AD^27^,^28^,^29^.

Given that mitochondrial dysfunction plays an important role in the pathogenesis of AD, early intervention with mitochondrial-targeted therapeutics, such as MitoQ^5^,^30^, to delay the progression of early AD could prove a promising treatment. Work aimed at exploiting our new nematode model for screening of putative AD therapeutics, especially early preventative treatment that specifically target mitochondria, is underway.

## Materials and Methods

### Nematode strains and general study design

The Bristol N2 (wild-type) strain were obtained from *Caenorhabditis* Genetics Center. Aβ_1–42_ minigene from ref. 8 was used, and cloned into the pTI11.1 plasmid which contains the *unc-119* promoter to drive pan-neuronal Aβ_1–42_ expression. Aβ_1–42_ minigene-containing pTI11.1 vector was co-injected with *myo-2::yfp* pharyngeal-specific fluorescence marker, both at a concentration of 25 ng/μl into the distal gonads of wild-type young adults. Ultraviolet (UV) irradiation was used to facilitate array integration into chromosomes. To exclude the possibility of unwanted mutation we outcrossed the strain four times, and independently created strains with the same construct. Those independently created strains showed similar behavioral and biochemical phenotypes. A vector control strain with only *myo-2::yfp* was generated alongside with the transgenic nematodes, and used as control for subsequent experiments. All strains were grown at 20 °C and maintained as previously described^14^.

All experiments used age-synchronized nematode obtained by hypochlorite bleaching. Adult age groups were categorized as follows: ‘young’, day 4 post-hatching, ‘middle age’, day 8 post-hatching, ‘old’, day 12 post-hatching. All behavioural studies were conducted under blinded conditions to minimize experimenter’s bias^31^. Except for lifespan and health-span studies, all other age-dependent experiments were carried out in the presence of 250 μM 5-fluoro-2′-deoxyuridine (Sigma-Aldrich, St. Louis, USA) to prevent progeny production. Unless otherwise indicated, all experiments were carried out in at least two independent cohorts, producing consistent results.

### RNA extraction and reverse transcription

Nematode pellets (200 nematodes) were snap frozen in liquid nitrogen and thawed three times. Total RNA was extracted using the RNeasy Micro Kit (Qiagen, Venlo, Netherlands), with DNase treatment, as per manufacturer’s instruction. cDNA was synthesized using Oligo-dT primer, with GoScript Reverse Transcription System (Promega, Madison, USA), as per manufacturer’s instruction.

### Real time polymerase chain reaction

For PCR using Taqman system, Taqman Universal PCR Master Mix was used with customized gene assays AJX00TP (Abeta) and Ce02507510_s1 (*act-2*: β-actin; Applied Biosystems, Foster City, USA) were used with the (Applied Biosystems, Foster City, USA). Cycling conditions were 50 °C × 2 min, then 95 °C × 10 min, followed by 40 cycles of 95 °C × 15 sec +60 °C × 1 min.

For PCR using SyBr system, QuantiFast SyBr Green PCR kit (Qiagen, Venlo, Netherlands) was used with self-design Abeta primers (FP: 5′ GAT GCA GAA TTT CGA CAT GAT TCA GG; RP: 5′ TCA AGC AAT GAC AAC TCC TCC C). Cycling conditions were 95 °C × 7 min, followed by 40 cycles of 95 °C × 10 sec + 60 °C × 1 min. All reactions were performed using a StepOne RT-PCR System (Life Technologies, Carlsbad, USA).

### DNA gel electrophoresis and sequencing

DNA fragments obtained from PCR were resolved on a 1.5% agarose gel at 100V for 80 min (Bio-Rad, Hercules, USA). DNA bands were visualized using an ECL imager (Thermo Fisher Scientific, Waltham, USA).

For sequencing, DNA samples obtained from PCR were purified using QIAquick PCR purification kit (Qiagen, Venlo, Netherlands). Single pass DNA sequencing was performed by Axil Scientific (1st BASE, Singapore, Singapore).

### Protein extraction and immunoblot analysis

Approximately 5000 nematodes were homogenized in 1X RIPA buffer (Cell Signaling, Beverly, USA) containing 1% protease inhibitor cocktail (Roche, Basel, Switzerland). After centrifugation, the supernatant was collected as the soluble fraction, while the pellet was re-extracted in 5M urea (Sigma-Aldrich, St. Louis, USA) containing 1% protease inhibitor cocktail (Roche, Basel, Switzerland). Ice sonication followed by centrifugation was performed to obtain the insoluble fraction.

Protein separation and immunoblotting were performed as described in ref. 32 with modifications. Modifications include probing for Aβ using anti-Aβ antibodies 6E10 (Covance Inc., California, USA) at a 1:1000 dilution and visualizing immune-positive Aβ bands by enhanced chemiluminescence using the SuperSignal West Femto Kit (Life Technologies, Carlsbad, USA). The membrane was stripped and reprobed with anti-α-tubulin (Sigma-Aldrich, St. Louis, USA) at a 1:3000 dilution for normalization.

### Lifespan, health-span and neuromuscular function scoring

Lifespan and health-span assays were conducted under blinded condition as described in ref. 33. Nematode motility was used as a marker of health-span, using the ABC scoring described in ref. 9. Nematodes with progeny that hatched internally were scored as bagging^10^. Nematodes that lost their dark-colored intestinal appearance and showed disorganized intestinal arrangement were scored as constipated^34^. For pharyngeal pumping assay, videos of nematodes were recorded at 80X magnification using a Leica IC80 HD microscope system. Number of pharyngeal pumps over a 10 sec time interval was then counted for each nematode. For head shake measurement, videos of nematodes were recorded at 10X magnification using a Leica IC80 HD microscope system. The average maximum angle of deviation from the central axis and the frequency of head movement from side-to-side were measured and their product was obtained as the sum of oscillatory angle.

### Behavioural assays

For the nematode loss assay, 25–30 nematodes were placed on 60 mm agar plates next to their food and incubated for 12 h. The percentage of nematodes that had crawled off the plates and had dried at the edge of plates was scored as lost. Food race assay was conducted as described in ref. 15 using 94 mm NGM plate.

### Biochemical assays

ATP luminescence assay^35^ was performed as described in ref. 33 using firefly lantern extract (Sigma-Aldrich, St. Louis, USA).

Mitochondrial metabolic flux was measured using XF96 Extracellular Flux Analyzer (Seahorse Bioscience, North Billerica, USA), as described in ref. 16.

For the measurement of ETC complex enzyme activities, isolation of mitochondria and measurement of Complex I and IV activities^36^,^37^ were performed as described in ref. 5 with approximately 5000 nematodes per replicate. This experiment was performed in only one cohort with three replicates, due to the large sample size needed.

### Statistical analyses

All statistical analyses were performed in GraphPad Prism (San Diego, USA). Difference between nematode strains was determined using two-way ANOVA analysis with post-tests for all age-related experiments, while an unpaired t-test was performed for non-age-related experiments. Values were reported as mean ± standard error or mean (SEM). P value of <0.05 was considered significant.

## Additional Information

**How to cite this article**: Fong, S. *et al.* Energy crisis precedes global metabolic failure in a novel *Caenorhabditis elegans* Alzheimer Disease model. *Sci. Rep.* **6**, 33781; doi: 10.1038/srep33781 (2016).

## Supplementary Material

Supplementary Information
