# Supplementary material for: Energy crisis precedes global metabolic failure in a novel Caenorhabditis elegans Alzheimer Disease model
Source: Sci Rep. 2016 Sep 22;6:33781. doi: 10.1038/srep33781 (PMC5031964; doi:10.1038/srep33781)
Supplement: Supplementary Information [file srep33781-s1.pdf]

Title: Energy crisis precedes global metabolic failure in a novel *Caenorhabditis elegans* Alzheimer Disease model

Author list: Fong Sheng[1*], Emelyne Teo[2*], Ng Li Fang[3], Chen Ce-Belle[4], Lakshmi Narayanan Lakshmanan[5], Tsoi Sau Yee[3], Philip Keith Moore[2], Takao Inoue[4], Barry Halliwell[4], Jan Gruber[3,4#]

[1]Internal Medicine Residency Programme, SingHealth Group, Singapore [2]NUS Graduate School for Integrative Sciences and Engineering, National University of Singapore, Singapore [3]Science Division, Yale-NUS College, Singapore [4]Department of Biochemistry, National University of Singapore, Singapore Institute of Chemical and Bioengineering, ETH Zurich, Switzerland

[*]Contributed equally to this work.

[#]Corresponding author. Email: bchjg@nus.edu.sg

Supplementary Tables

Table S1. Transgenic *C. elegans* strains expressing human Aβ

Strain	Type of expression	Location of expression	Type of Aβ	Reference
CL2006	Constitutive	Muscle	$A\beta_{3-42}$	Link 1995
CL4176	Inducible (by temperature up-shift)	Muscle	$A\beta_{3-42}$	Link et al. 2003
GMC101	Inducible (by temperature up-shift)	Muscle	$A\beta_{1-42}$	McColl et al. 2012
CL2355	Inducible (by temperature up-shift)	Neuron	$A\beta_{3-42}$	Link 2005
FDX (ses25)	Constitutive	ASE (chemosensory neuron)	$A\beta_{3-42}$	Duan & Sesti 2013
UA166	Constitutive	Glutaminergic neuron	$A\beta_{3-42}$	Treusch et al. 2012
Our strain	*Constitutive*	*Neuron*	$A\beta_{1-42}$	*Fong et al. 2015*

Table S2. Summary of lifespan studies conducted.

Study	Median LSP (days)	Log-rank test	Mean LSP (days)	t-test	Max LSP (days)	t-test	N
1	Control: 17<br>Aβ: 13	**P<0.001**	Control: 15.9<br>Aβ: 13.4	**P<0.001**	Control: 22.8<br>Aβ: 19.9	**P<0.01**	Control: 168<br>Aβ: 122
2	Control: 18<br>Aβ: 13	**P<0.001**	Control: 17.4<br>Aβ: 13.3	**P<0.001**	Control: 25.5<br>Aβ: 22.1	**P<0.001**	Control: 137<br>Aβ: 107
3	Control: 17<br>Aβ: 12	**P<0.001**	Control: 16.7<br>Aβ: 12.1	**P<0.001**	Control: 24.2<br>Aβ: 19.1	**P<0.001**	Control: 88<br>Aβ: 88

Supplementary Figures

A)

Sequences used in this study:

Nucleotide

```
ggtacc atg cat aag gtt ttg ctg gca ctg ttc ttt atc ttt
       ctg gca cca gca gga aca GAT GCT GAT GCA GAA TTT
       CGA CAT GAT TCA GGA TAT GAA GTT CAT CAT CAA AAA
       TTG GTG TTC TTT GCA GAA GAT GTG GGA TCA AAT AAA
       GGA GCA ATT ATT GGA CTC ATG GTG GGA GGA GTT GTC
       ATT GCT TGA actagt
```

Amino acid (predicted) – residues 1 – 42 of human Aβ

```
kpnI - met-his-lys-val-leu-leu-ala-leu-phe-phe-ile-phe-
       leu-ala-pro-ala-gly-thr-ASP-ALA-ASP-ALA-GLU-PHE-
       ARG-HIS-ASP-SER-GLY-TYR-GLU-VAL-HIS-HIS-GLN-LYS-
       LEU-VAL-PHE-PHE-ALA-GLU-ASP-VAL-GLY-SER-ASN-LYS-
       GLY-ALA-ILE-ILE-GLY-LEU-MET-VAL-GLY-GLY-VAL-VAL-
       ILE-ALA-stop - speI
```

B)

C. elegans Genetic Code:

TTT	Phe	0.50	TCT	Ser	0.21	TAT	Tyr	0.56	TGT	Cys	0.55
TTC	Phe	0.50	TCC	Ser	0.13	TAC	Tyr	0.44	TGC	Cys	0.45
TTA	Leu	0.12	TCA	Ser	0.25	TAA	Stop	0.44	TGA	Stop	0.39
TTG	Leu	0.23	TCG	Ser	0.15	TAG	Stop	0.17	TGG	Trp	1.00
CTT	Leu	0.24	CCT	Pro	0.18	CAT	His	0.61	CGT	Arg	0.21
CTC	Leu	0.17	CCC	Pro	0.09	CAC	His	0.39	CGC	Arg	0.10
CTA	Leu	0.09	CCA	Pro	0.53	CAA	Gln	0.66	CGA	Arg	0.23
CTG	Leu	0.14	CCG	Pro	0.20	CAG	Gln	0.34	CGG	Arg	0.09
ATT	Ile	0.53	ACT	Thr	0.33	AAT	Asn	0.62	AGT	Ser	0.15
ATC	Ile	0.31	ACC	Thr	0.18	AAC	Asn	0.38	AGC	Ser	0.10
ATA	Ile	0.16	ACA	Thr	0.34	AAA	Lys	0.59	AGA	Arg	0.29
ATG	Met	1.00	ACG	Thr	0.15	AAG	Lys	0.41	AGG	Arg	0.08
GTT	Val	0.39	GCT	Ala	0.36	GAT	Asp	0.68	GGT	Gly	0.20
GTC	Val	0.22	GCC	Ala	0.20	GAC	Asp	0.33	GGC	Gly	0.12
GTA	Val	0.16	GCA	Ala	0.31	GAA	Glu	0.62	GGA	Gly	0.59
GTG	Val	0.23	GCG	Ala	0.13	GAG	Glu	0.38	GGG	Gly	0.08

Fields: Triplet | Amino acid | Fraction

C)

```
1    GATGCAGAATTTCGACATGATTCAGGATATGAAGTTCATCATCAAAAATTGGTGTTCTTT  60
     ||||||||||||||||||||||||||||||||||||||||||||||||||||||||||||
1    GATGCAGAATTTCGACATGATTCAGGATATGAAGTTCATCATCAAAAATTGGTGTTCTTT  60

61   GCAGAAGATGTGGGATCAAATAAAGGAGCAATTATTGGACTCATGGTGGGAGGAGTTGTC  120
     ||||||||||||||||||||||||||||||||||||||||||||||||||||||||||||
61   GCAGAAGATGTGGGATCAAATAAAGGAGCAATTATTGGACTCATGGTGGGAGGAGTTGTC  120

121  ATTGCTTGA  129
     |||||||||
121  ATTGCTTGA  129
```

Figure S1. A) Aβ$_{1-42}$ nucleotide sequence used in minigene construction. Additional nucleotides, gatgct, encoding aspartate and alanine, at the N-terminus, to prevent formation of truncated Aβ$_{3-42}$. **B) *C. elegans* genetic code frequency.** Codon usage was optimized by using nematode codons with highest frequencies to encode the Aβ$_{1-42}$ minigene. **C) Sequence alignment of DNA identity obtained from sequencing.** The Aβ sequence detected from the transgenic nematodes matches with the codon optimized full length Aβ$_{1-42}$ nucleotide sequence.
